# Computational Prediction of Effector Proteins in Fungi: Opportunities and Challenges

**DOI:** 10.3389/fpls.2016.00126

**Published:** 2016-02-12

**Authors:** Humira Sonah, Rupesh K. Deshmukh, Richard R. Bélanger

**Affiliations:** Département de Phytologie, Faculté des Sciences de l’Agriculture et de l’Alimentation, Centre de Recherche en Horticulture, Université Laval, QuébecQC, Canada

**Keywords:** computational tool and servers, classification and prediction, effector proteins, fungal secretome, host–pathogen interaction

## Abstract

Effector proteins are mostly secretory proteins that stimulate plant infection by manipulating the host response. Identifying fungal effector proteins and understanding their function is of great importance in efforts to curb losses to plant diseases. Recent advances in high-throughput sequencing technologies have facilitated the availability of several fungal genomes and 1000s of transcriptomes. As a result, the growing amount of genomic information has provided great opportunities to identify putative effector proteins in different fungal species. There is little consensus over the annotation and functionality of effector proteins, and mostly small secretory proteins are considered as effector proteins, a concept that tends to overestimate the number of proteins involved in a plant–pathogen interaction. With the characterization of *Avr* genes, criteria for computational prediction of effector proteins are becoming more efficient. There are 100s of tools available for the identification of conserved motifs, signature sequences and structural features in the proteins. Many pipelines and online servers, which combine several tools, are made available to perform genome-wide identification of effector proteins. In this review, available tools and pipelines, their strength and limitations for effective identification of fungal effector proteins are discussed. We also present an exhaustive list of classically secreted proteins along with their key conserved motifs found in 12 common plant pathogens (11 fungi and one oomycete) through an analytical pipeline.

## Introduction

The importance of fungi as plant pathogens has spurred scientists to study their biology. Fungal pathogens cause enormous yield losses in agricultural crops and post-harvest products (Dean et al., 2012). Generally, the losses caused by pests and diseases are considered to be 20–40% of the total production, and the resulting consequences on human health, the world economy, environmental and ecological losses are significant factors to be considered (Savary et al., 2012; Balint-Kurti and Holland, 2015). To prevent such losses, the use of resistance genes and the application of fungicides are the two major options available for the farmers (Delourme et al., 2006; Dean et al., 2012; Sonah et al., 2012). In the latter case, fungal pathogens are known to quickly develop resistance to most chemicals and the use of fungicides is generally perceived as negative for human health and the environment (Van de Wouw et al., 2014; Balint-Kurti and Holland, 2015). For this reason, genetic approaches are considered safer and more durable, and considerable efforts are deployed toward the identification and introgression of resistance genes into plant material (Channamallikarjuna et al., 2010; Raman et al., 2012; Singh et al., 2012; Saha et al., 2014). However, the use of a single source of resistance also brings tremendous selection pressure on the pathogen, and the resistance often breaks down quite rapidly (Kutcher et al., 2013; Van de Wouw et al., 2014). For instance, resistance breakdown to the blackleg disease in canola crops has been reported recently in Australia (Van de Wouw et al., 2014). To achieve more durable resistance against a wide range of fungal pathogen races, a thorough understanding of the virulence factors released by the pathogen and the resulting plant immune responses is a prerequisite.

Fungi have adopted diverse strategies to interact with host plants and to overcome a complex network of plant defense mechanisms. The first line of defense involves recognition of the pathogen based on conserved molecular features generally known as pathogen-associated molecular patterns (PAMPs; Silva-Gomesa et al., 2014). The PAMPs, like chitin or glucan residues of fungi, are recognized by plant receptors known as pattern recognition receptors (PRRs). PRRs recognize PAMPs and induce PAMP triggered immunity (PTI) through the secretion of antifungal compounds, production of reactive oxygen species (ROS), phytoalexins, protease inhibitors, chitinases and glucanases. In turn, to overcome PRR responses, pathogens secrete effector molecules, which can lead to plant effector-triggered immunity (ETI; Giraldo and Valent, 2013). The functional and structural alterations in plants caused by effector molecules either facilitate infection by the pathogen through release of virulence factors and toxins, or trigger defense responses based on recognition of avirulence factors and elicitors, or both (Jones and Dangl, 2006; Kamoun, 2006; Morgan and Kamoun, 2007). The effectors are recognized by the specific resistance gene(s) mostly coding for proteins having interactive domains, such as the NB-LRR protein that induces the ETI in plants. Natural selection of pathogens against the resistance pressure applied by ETI involves diversifying unrecognizable effectors (Jones and Dangl, 2006). Such co-evolution of genes involved in plant–pathogen interactions has been previously described by Jones and Dangl (2006) in the form of the simplified and understandable “Zigzag model.” The zigzag model can be summarized with four stages: in the first stage, PRRs recognize PAMPs; in the second stage, to overcome PRR responses, pathogens secrete effectors to interfere with PTI; in the third stage, NB-LRRs recognize effectors; and finally in the fourth stage, diversification and loss or gain of effectors lead to co-evolution.

The genes coding for effectors are mostly known as *Avr* genes and the complementary trigger-coded responses by the host are denoted as *R* genes. The ETI involves the hypersensitive response (HR) that restricts pathogen growth. Evolutionary changes in effector (*Avr*) genes make them unrecognizable by the host *R* genes resulting in a compatible interaction, or disease. Since *Avr* genes evolve quickly, they can overcome the plant defense mechanisms within a short period of time. Therefore, effectors are important targets to consider in attempts to enhance plant immunity against pathogens.

## Characteristics of Effector Proteins

The definition of effector is constantly evolving with the increased understanding of the molecular mechanisms involved in pathogenicity. At times, plant pathologists will use the term effector in a broader sense including all molecules, like proteins, carbohydrates, and secondary metabolites, potentially involved in the infection process. Based on a broader definition, PAMPs can also be referred to as effectors (Kamoun, 2006; Nemri et al., 2014).

**FIGURE 1:**
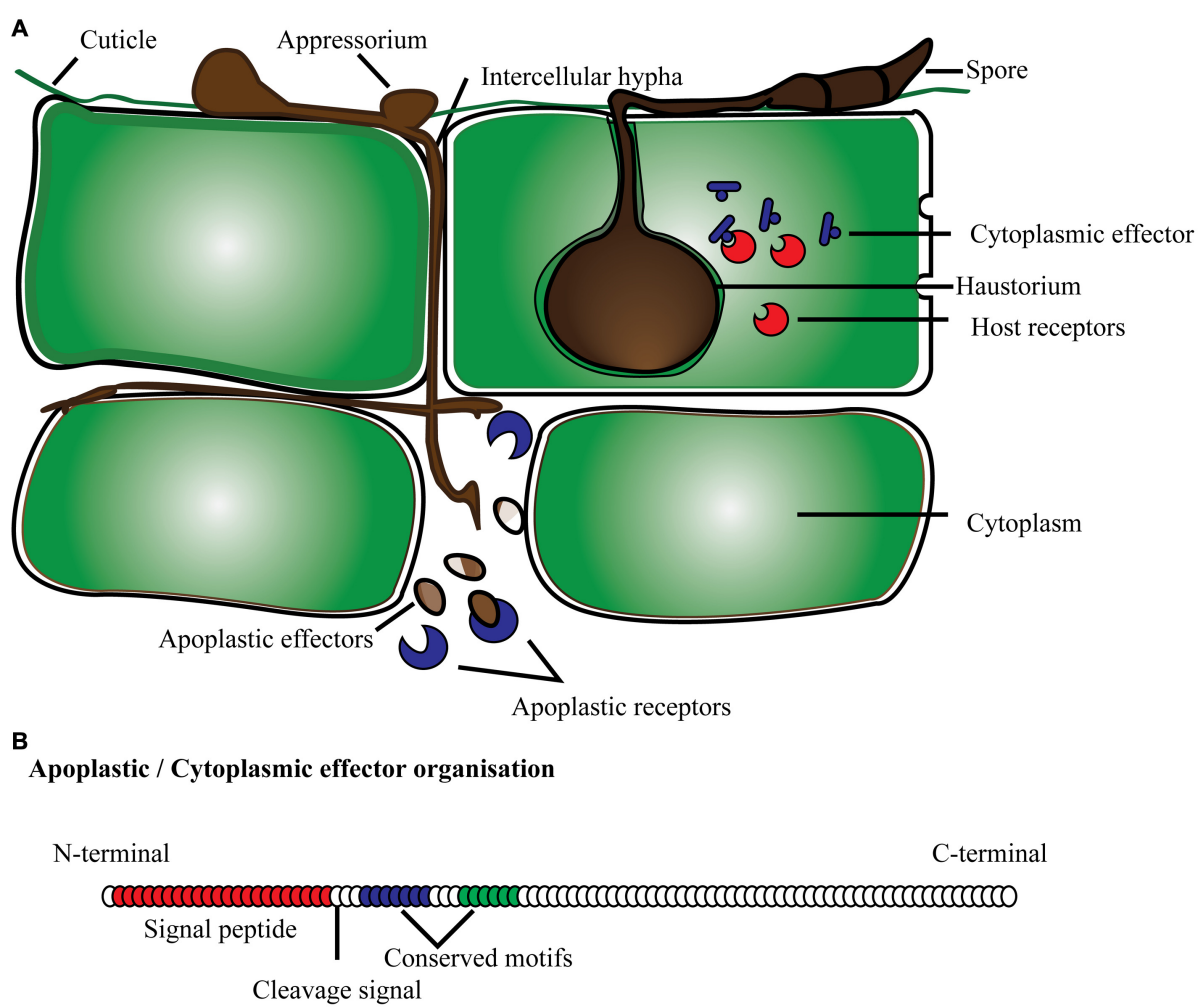
**Schematic representation of effector proteins **(A)** secreted by fungi/oomycetes in the cytoplasmic and apoplastic region of the plant cell; **(B)** typical protein organization of apoplastic and cytoplasmic effectors with signal peptide, cleavage site and conserved domain present toward the N-terminus**.

Effector proteins are mostly secretory proteins that alter host cells to suppress host defense mechanisms, and facilitate infection by the pathogen so it can derive nutrients from the host. Effectors may also activate defense strategies in resistant plant genotypes. Criteria to fit the definition of candidate secreted effector proteins (CSEPs) include: fungal proteins with a signal peptide for secretion, no trans-membrane domains, no similarity with other obvious protein domains, fairly small size and mostly species-specific (Jones and Dangl, 2006; Stergiopoulos and de Wit, 2009; Djamei et al., 2011; Lo Presti et al., 2015). In general, effector proteins are modular proteins. Expression of effector proteins follows contact with the host tissue and it is very specific with different stages of disease development. Fungal pathogens have evolved the capacity to deliver effector proteins inside the host cell through diverse mechanisms (**Figure 1**). They can secrete effector proteins inside the host cytoplasm as well as in the extracellular space, and are subsequently classified as cytoplasmic and apoplastic effectors, respectively. The standard protein organization of apoplastic effectors contains a signal peptide within the initial 60 amino acids (AA) at the N terminus followed by multiple domains toward the C terminus. These types of effectors are comparatively small, and rich in cysteine residues like most of the serine or cysteine protease inhibitor proteins. For instance, known effectors of the tomato fungal pathogen *Cladosporium fulvum* such as Avr2, Avr9, Avr4, and ECP2, are small cysteine-rich proteins that are thought to function exclusively in the apoplast (Thomma et al., 2005). The apoplastic effectors of *C. fulvum*, and other fungal and oomycete pathogens have the ability to inhibit and protect against plant hydrolytic enzymes, such as proteases, glucanases, and chitinases (reviewed by Misas-Villamil and van der Hoorn, 2008). Another example is effector protein SnTox1 identified in the fungal pathogen *Stagonospora nodorum*, which consists of 117 amino acids with the first 17 predicted as a signal peptide and 16 of the remaining 100 amino acids being cysteine residues (Liu et al., 2012). Similarly, cytoplasmic effectors have a secretion signal at the N terminus, and multi-domain toward the C terminus. In addition, conserved amino acid motifs specific to effectors have been reported, namely in oomycetes (Morgan and Kamoun, 2007; Jiang et al., 2008; Ye et al., 2015). The most common motif, RxLR (arginine, any AA, leucine, arginine), has been identified in over 700 CSEPs predicted in two *Phytophthora* species, *P. sojae* and *P. ramorum* (Jiang et al., 2008). The majority of RxLR carrying effectors also possess a second conserved motif termed dEER (aspartate, glutamate, glutamate, arginine), which is present toward the C-terminus. Similarly, with the increased number of predicted CSEPs, more conserved features may be discovered. A comparative analysis of *Phytophthora* CSEPs has identified three more conserved motifs denoted as W, Y and L toward the C-terminus (Jiang et al., 2008; Win et al., 2012; Wirthmueller et al., 2013). These domains form an alpha-helical fold termed WY fold that is supposed to provide a structure flexibility leading toward the surface diversification of RxLR effectors (Win et al., 2012; Wirthmueller et al., 2013).

The effector protein family encompassing the RxLR motif is found to be the largest among oomycete CSEPs. Even with such a common conserved motif, this CSEP family is very diverse mostly because of high positive selection pressure. Recently, secondary structure analyses of the RxLR effectors have identified abundant short alpha-helices at the C-terminus in the majority of proteins (Ye et al., 2015). Similarly, de Guillen et al. (2015) have observed common 3-dimensional structures despite a lack of sequence similarity among the AVR1-CO39 and AVR-Pia effectors of *Magnaporthe oryzae*. Structural similarity searches have also succeeded to identify two more effectors, one each from *M. oryzae* (AvrPiz-t), and *Pyrenophora tritici-repentis* (ToxB; de Guillen et al., 2015). The identification of similar secondary or tertiary structures may represent another promising approach to identify functional effectors. The abundant short alpha-helices have also been confirmed in the previously characterized RxLR effectors including PcAvr3a4, PcAvr3a11, PsAvh5, PexRD2, HaATR1, and HaATR13, and also observed in effectors lacking RxLR (Boutemy et al., 2011; Chou et al., 2011; Yaeno et al., 2011; Sun et al., 2013; Ye et al., 2015). The RxLR motif is found to be more common in oomycetes particularly in *Phytophthora* species but is also found, albeit in reduced numbers, in other oomycetes and even in fungal species (Morgan and Kamoun, 2007; Jiang et al., 2008; Ye et al., 2015). This suggests that fungi might contain other functionally important motifs like RxLR, but with a relatively lower frequency, which makes it difficult to identify based on the degree of conservation. For instance, a highly conserved pattern of seven amino acids “RSIDELD” at the C-terminus (named DELD) has been identified in 25 CSEPs of *Piriformospora indica* (root endophyte; Zuccaro et al., 2011). A total of 107, 178, and 57 CSEPs have been identified in powdery mildew of barley, stem rust, and leaf rust of wheat, respectively, with a conserved motif of three AA in which the first AA is aromatic like tyrosine, phenylalanine or tryptophan, and the last is always a cysteine (Y/F/WxC; Godfrey et al., 2010; Pedersen et al., 2012). This finding suggests that the Y/F/WxC motif containing CSEPs constitutes a new class of effectors that could denote specificity to haustoria-producing pathogenic fungi.

## Computational Tools and Pipelines Available for Prediction of Candidate Secretory Effector Proteins

Many studies employing computational prediction of CSEPs followed by identification of conserved motifs lack experimental validation of the results (Godfrey et al., 2010; Zuccaro et al., 2011; Ye et al., 2015). Nevertheless, computational prediction serves as an excellent starting point to screen CSEPs for functional analysis and also helps to understand the evolution, distribution and characterisation of effectors.

**FIGURE 2:**
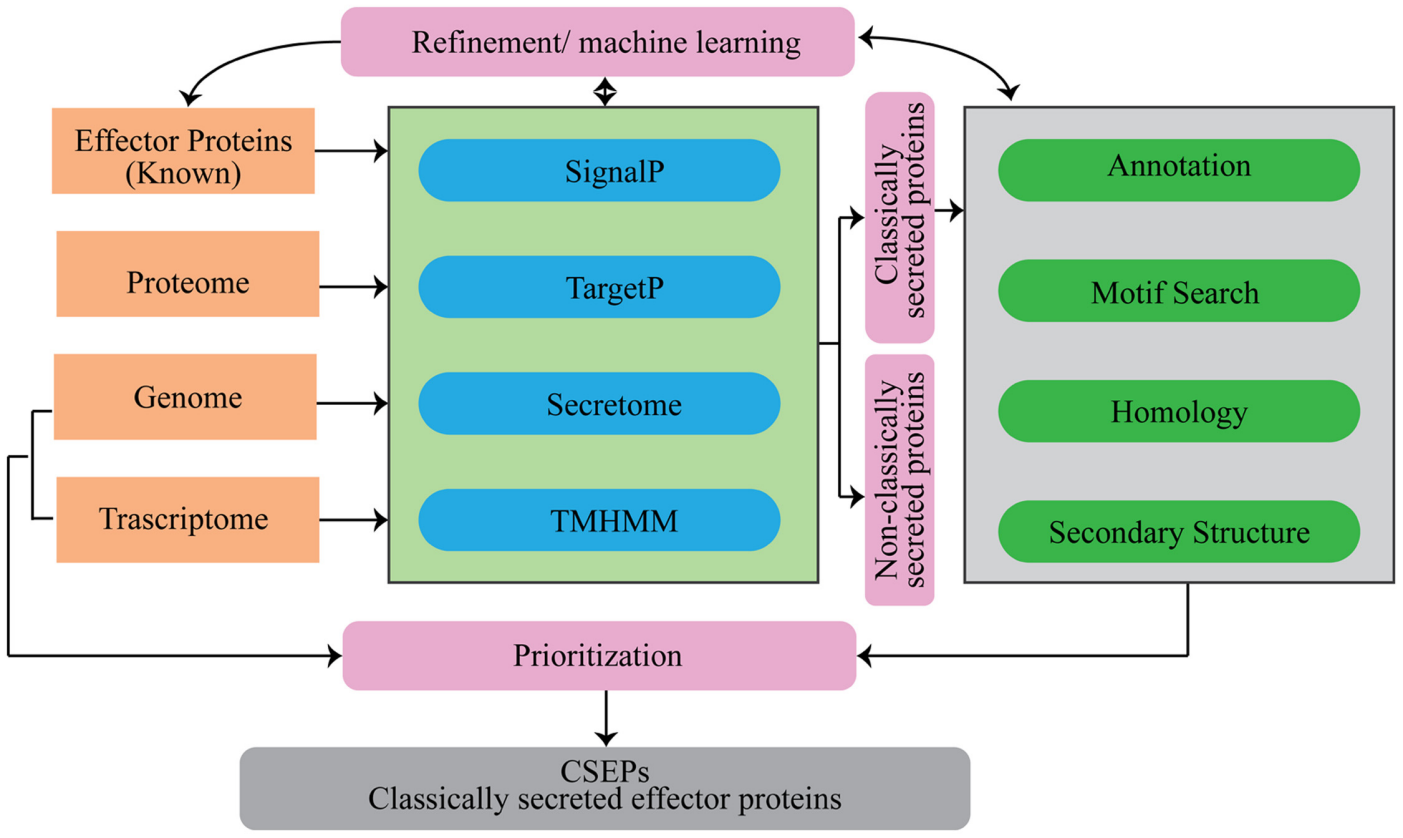
**Flowchart of analytical tools that can be used for the prediction of secretome and candidate secretory effector proteins (CSEPs) in fungi**.

Several computational tools and web servers are available for the characterization of proteins using the AA sequence as an input. In the case of CSEP prediction, computational tools have been used to systematically sort the list based on some basic pre-established criteria (**Figure 2**).

### Signal Peptide

Commonly, the first step of the CSEP prediction is to look for the extracellular secretion signals. Eukaryotic as well as prokaryotic proteins usually contain a signal peptide that guides their translocation across the membranes. As a general rule, signal peptides are 20–30 AA in length and they have a positively charged N-terminus, followed by a hydrophobic region, and cleavage site at the C-terminus. In spite of these unique properties, there is limited sequence homology or similarity among signal peptides. Therefore, routine BLAST search alone is not useful for signal peptide prediction and it requires complex analytical algorithms like neural networks, machine learning systems, and Hidden Markov model (HMM). There are several computational tools available that use a combination of different sophisticated algorithms and generally have a very high sensitivity and accuracy for predicting signal peptides (**Table 1**).

**Table 1:** Features of important tools available for the identification of secretory proteins in fungi and other eukaryotes.

Tool (specification)	Important features	Reference/website
SignalP server	Predicts signal peptide cleavage sites in proteins from different organisms including eukaryotes. Prediction is based on a combination of several artificial neural networks	Petersen et al., 2011 www.cbs.dtu.dk/services/SignalP
Philius server	Predicts topology and signal peptides using dynamic Bayesian networks	Reynolds et al., 2008 http://www.yeastrc.org/philius
SPOCTOPUS server	Combines prediction of signal peptides and membrane protein topology, suitable for genome-scale studies	Viklund et al., 2008 http://octopus.cbr.su.se/
SPSCAN	Predicts secretory signal peptides in protein sequences. For each sequence, provides secretory signal peptides sorted according to score	http://www.csd.hku.hk/bruhk/gcgdoc/spscan.html
Phobius web server	Combines transmembrane (TM) topology and signal peptide predictions and provides optimal choice between TM segments and signal peptides	Käll et al., 2007 http://phobius.binf.ku.dk
ProtComp	Uses neural networks-based prediction; direct comparison with known homologous proteins; comparisons of pentamer distributions calculated for query and DB sequences; prediction of signal peptides, signal-anchors, GPI-anchors, transit peptides of mitochondria, and TM segments	http://linux1.softberry.com
PrediSi (PREDIction of SIgnal peptides)	Uses position weight matrix approach that is improved by a frequency correction that considers amino acid bias present in proteins. The tool is also trained using a large number of sequences from SwissProt database	Hiller et al., 2004 http://www.predisi.de/home.html
Signal-CF	Uses automated methods that first predict secretory or non-secretory proteins and further identify the cleavage site of the signal peptide	Chou and Shen, 2007 http://chou.med.harvard.edu/bioinf/Signal-CF/
Signal-3L	Consists of three prediction engines for identification of secretory or non-secretory proteins by OET-KNN and PseAA; predicts signal peptide cleavage sites by a subsite-coupled discrimination algorithm; and determines the final cleavage site by fusing the global sequence alignment outcome	Shen and Chou, 2007 http://chou.med.harvard.edu/bioinf/Signal-3L/
WoLF PSORT	Converts protein amino acid sequences into numerical localization features; based on sorting signals, amino acid composition and functional motifs to predict protein subcellular location	Horton et al., 2007 www.wolfpsort.org


### Transmembrane Domains

Distinguishing a secretory protein from a transmembrane (TM) protein is difficult since both have hydrophobic segments. In the case of TM proteins, the hydrophobic segment is usually longer than in the secretory proteins. Therefore, to avoid false positive prediction of secretory proteins, it is always necessary to identify TM domains in candidate proteins. As with signal prediction tools, TM domain prediction tools also use complex algorithms. There are several online tools and web-servers available for the purpose of predicting TM-domains (**Table 2**). To make prediction of secretory proteins, more sophisticated tools like ProtComp, Phobius, and SPOCTOPUS hosts combine algorithms for TM-domain and signal peptide prediction. Proteins having signal peptides for secretion are not systematically secreted, since some of them may be anchored in the endoplasmic reticulum due to the hydrophobic signal at the C terminus, or the presence of one or more TM domains. Similarly, proteins with glycosylphosphatidylinositol (GPI) anchors stay inserted in the membrane since they have glycolipids attached to the C-terminus (Petersen et al., 2011). Therefore, during secretome analysis, it is always better to predict features like signal-anchors, GPI-anchors, and transit peptides of plastids along with signal peptides and TM-domains for an effective characterisation of CSEPs.

**Table 2:** Features of the most common computational tools available for the prediction of trans-membrane (TM) domains.

Tools	Important features	Website/reference
TMHMM Server v. 2.0	Predicts trans-membrane (TM) domains; a common mistake by the program consists in reversing the direction of proteins with one TM segment	www.cbs.dtu.dk/services/TMHMM/
TMpred	Predicts TM regions and orientation. The TMpred program makes a prediction of membrane-spanning regions and their orientation	www.ch.embnet.org
		Hofman, 1993
TMbase	Offers a good database of TM proteins and their helical membrane- spanning domains; TMbase was originally meant as a tool for analyzing the properties of TM proteins	www.ch.embnet.org
		Hofman, 1993
HMMTOP	Serves as automatic server for predicting TM helices and topology of proteins	Tusnady and Simon, 2001 www.enzim.hu/hmmtop/
PredictProtein	Predicts secondary structures	Rost et al., 2004 www.predictprotein.org/
SOSUI	Classifies and predicts secondary structures of membrane proteins	http://harrier.nagahama-i-bio.ac.jp/sosui/
TopPred 1.10	Predicts topology of membrane proteins	http://mobyle.pasteur.fr/cgi-bin/portal.py?\#forms::toppred
DAS-TMfilter server	Filters false positive TM protein predictions	http://mendel.imp.ac.at/sat/DAS/DAS.html
CCTOP (Consensus Constrained TOPology prediction)	Performs TM topology predictions	http://cctop.enzim.ttk.mta.hu/
MetaTM	Predicts TM topologies through a consensus method	Klammer et al., 2009 http://metatm.sbc.su.se/
MINNOU	Predicts membrane proteins with and without explicit use of hydropathy profiles and alignments	http://minnou.cchmc.org/
PHDhtm	Predicts the location of helical TM segments in integral membrane proteins through a neural network system	https://npsa-prabi.ibcp.fr/cgi-bin/npsa_automat.pl?page=/NPSA/npsa_htm.html
Phobius	Combines TM topology and signal peptide predictions	Käll et al., 2007
		http://phobius.sbc.su.se/poly.html
PRED-TMR	Refines a standard hydrophobicity with a detection of potential edges	Pasquier et al., 1999
		http://athina.biol.uoa.gr/
SCAMPI	Uses position-specific amino acid contributions to the free energy of membrane insertion and also uses the current best statistics-based topology predictors	Bernsel et al., 2008 http://scampi.cbr.su.se/
SOMRuler	Offers an interpretable TM helices predictor	Yu et al., 2011 www.csbio.sjtu.edu.cn/bioinf/SOMRuler/
ConPred	Predicts TM topology based on a consensus approach by combining the results of several methods	Arai et al., 2004
		http://bioinfo.si.hirosaki-u.ac.jp/ConPred2/
TMBB-DB	Compiles the predictions made by the Freeman–Wimley algorithm	http://beta-barrel.tulane.edu/
TMalphaDB	Quantifies the structural distortion induced by a sequence motif in alpha TM segments	http://lmc.uab.cat/TMalphaDB/
TMexpo	Predicts rotational preferences of TM helices to facilitate structural modeling. TMexpo calculates rotational angles of TMHs based on the predicted relative accessible surface area	http://bio-cluster.iis.sinica.edu.tw/TMexpo/
TMMOD	Uses an improved hidden Markov model for the identification and topology prediction of TM proteins	http://liao.cis.udel.edu/website/servers/TMMOD/scripts/frame.php?p=submit
Asymmetric Ez	Assesses the energy and positions of protein sequences or structures in and on the membrane through a knowledge-based potential	http://ez.degradolab.org/ez/


### Secretome

The entire secretome is expectedly not confined to disease-related proteins, and therefore, it needs to be sorted using features that are more specific to CSEPs. To apply different CSEP-specific criteria, several tools need to be applied in a systematic manner. The sequential use of different computation tools to obtain the desired outcome is known as an analytical pipeline. The literature offers a number of analytical pipelines for the identification of CSEPs. Notably, a pipeline based on HMM analyses followed by unsupervised protein clustering has been developed and implemented for the identification of 2830 CSEPs in the cereal pathogen *Fusarium graminearum* (Sperschneider et al., 2013). This pipeline has successfully identified CSEPs, conserved patterns and fungal motifs related to pathogenesis. Similarly, a pipeline developed by Saunders et al. (2012) proposes general basic features expected for the effective identification of CSEPs in rust fungi. The pipeline incorporates six major steps including secretome prediction, grouping of secreted and non-secreted proteins based on Markov clustering, functional annotation based on homology searches, searches for conserved motifs, effector features annotation, and finally hierarchical tribe clustering to rank and classify CSEPs (Saunders et al., 2012). The final ranking based on the fulfillment of different criteria is very helpful for the prioritization of candidates for functional characterization. In addition, understanding of the secondary and tertiary structure organization of effectors and their counterpart R genes will definitely improve the efficiency of computational tools to identify effectors more precisely (de Guillen et al., 2015; Maqbool et al., 2015; Ye et al., 2015).

## Different Conserved Motifs Identified in Fungal Genomes with Computational Mining

Amino acid sequences of functionally important motifs in CSEPs appear to be conserved across the fungal/omycete species. Therefore to understand the function of a given protein, analysis of such conserved motifs is required. Several reports have identified conserved motifs in effectors, namely in oomycetes, and validated their functionality (Morgan and Kamoun, 2007; Jiang et al., 2008; Godfrey et al., 2010; Zuccaro et al., 2011). The conserved motifs are found to play an important role in delivering effector proteins more efficiently during pathogenesis (Kale and Tyler, 2011; Petre and Kamoun, 2014). Natural variants of motif sequences, or variants created using mutagenesis, have been routinely evaluated with different approaches to confirm the functional role of the motifs. Plant transient-expression systems, in which candidate effectors are expressed in the plant and the translated protein observed for its secretion and re-entry into the plant cell, are commonly used to demonstrate the functional role of a motif and/or an effector (Kale and Tyler, 2011). Another approach consists in the application of purified effector proteins to leaf or root segments, where the entry of proteins into the cell is observed with the help of fluorescent peptide tags or by the use of antibodies (Kale and Tyler, 2011; Tanaka et al., 2015).

Several conserved motifs observed in oomycetes have also been found in different fungal genomes (**Table 3**). A systematic similarity search performed in secretomes of 11 fungi and one oomycete species, representing some of the most devastating plant pathogens, has shown the presence of different conserved motifs (**Table 3**, **Supplementary Table S1**, **Supplementary Figure S1**). Most of the conserved motifs identified to date, such as RxLR and DEER are small in length. Consequently, there are more chances to identify false positives of such motifs when using a similarity-based search. For example when we performed a similarity search using the FEMO software tool with an E-value cut-off at 0.001 (Grant et al., 2011), we found four times more CSEPs with a RxLR motif in *Magnaporthe grisea* than we did by using a more stringent cut-off at 0.0001 (**Table 3**, **Supplementary Table S2**). By using similar stringent conditions, we still observed the presence of the RxLR motif in all fungal secretomes studied, although with a considerably lower number than in *Phytophthora infestans.* The presence of a functional RxLR motif in a fungal genome has been debated since it is not as abundant as in the oomycetes. However, effector re-entry assays performed with Avr2 (*Fusarium oxysporum*) and AvrLm6 (*Leptosphaeria maculans*) have shown loss of functionality when mutations were made in RxLR-like motifs (Kale et al., 2010; Kale and Tyler, 2011). This suggests that RxLR-like motifs, in spite of their low occurrence, have a functional role in fungal effectors, and similar findings are expected for other motifs like DEER, [KRHQSA][DENQ]EL, [Y/W]xC, and RSIVEQD. Interestingly, unlike RxLR, we found that the motif RxLx[EDQ] occurred with a similar frequency in both fungal and oomycete secretomes (**Table 3**).

**Table 3:** Number of proteins, classically and small secreted proteins, and proteins bearing known conserved motifs identified in the genomes of 11 fungal and oomycete pathogens of crop plants.

S.No.	Species^†^	Total proteins	Classically secreted proteins^#^	Small secreted proteins^∗^	Known conserved motif^§^					
	
					RXLR	DEER	RXLX [EDQ]	[KRHQSA] [DENQ]EL	[Y/W]XC	RSIVEQD
1	*Leptosphaeria maculans*	12469	552	263	12	3	16	8	22	14
2	*Magnaporthe oryzae*	12755	983	528	43	2	22	16	43	27
3	*Ustilago maydis*	6522	358	142	21	0	13	13	5	10
4	*Puccinia graminis f. sp. tritici*	15979	886	612	17	2	13	14	43	14
5	*Cladosporium fulvum*	14127	711	296	17	3	17	17	20	19
6	*Fusarium oxysporum*	17696	824	361	17	3	19	17	44	30
7	*Mycosphaerella graminicola*	10933	518	235	14	1	6	8	22	20
8	*Colletotrichum graminicola*	12020	829	352	19	2	20	20	39	20
9	*Blumeria graminis*	6470	247	143	5	0	3	6	7	2
10	*Alternaria brassicicola*	10688	508	228	11	3	12	19	23	7
11	*Pyrenophora tritici-repentis*	12169	669	328	12	6	10	18	33	17
12	*Phytophthora_infestans* (Oomycete)	18140	671	343	74	22	16	10	18	11


*^†^Details of protein sequences retrieved from different databases are provided in **Supplementary Table S1**; ^#^The analytical pipeline (**Supplementary Figure S1**) was used for the identification of classically secreted proteins (Cortázar et al., 2014); ^∗^Classically secreted proteins having less than 300 amino acid length; ^§^Previously known Motif was used as query to perform similarity based motif-search using FIMO software tool implemented in MEME suite (Grant et al., 2011), To claim a significant match, an E-value cut-off at 0.0001 was used.*

## Secretory Proteins and Candidate Secretory Effector Protein (CSEP) Databases

Numerous accessible online databases have been developed to provide a catalog of well-characterized predicted secretory proteins and publically available CSEPs (**Table 4**). For instance, the Fungal Secretome Database (FSD) comprises predicted secretory proteins from 158 fungal/oomycete genomes. FSD relies on nine different prediction programs to build its inventory, namely SignalP 3.0, SigCleave, SigPred, RPSP, TMHMM 2.0c, TargetP 1.1b, PSort II, SecretomeP 1.0, and predictNLS (Choi et al., 2010). This secretome resource is very useful to identify and characterize species-specific conserved motifs. For instance, 734 putative RxLR effectors have been identified from three *Phytophthora* species, data that are well-correlated with those previously reported by Jiang et al. (2008) in the same species. Interestingly, the RxLR motif was observed with a very low frequency (0.04%) in the other 153 fungal genomes (Choi et al., 2010). This finding is surprising since many more fungal genomes have been observed to have a much higher number of RxLR and RxLR-like effectors (**Table 3**). While there is no doubt that the RxLR motif is more abundant and conserved in oomycetes, and more particularly in *Phytophthora* species, these observations raise interesting questions about the evolution, transfer specificity and functionality of RxLR effectors.

**Table 4:** Features of databases available for effectors, secreted proteins and virulence factors identified in fungal genomes.

Database (specificity)	Important features	Reference/website
DFVF (Database of fungal virulence factors)	Comprises host fungal virulence factors, including 2058 pathogenic genes produced by 228 fungal strains from 85 genera	http://sysbio.unl.edu/DFVF/
		Lu et al., 2012
FSD (Integrated platform for annotation of fungal secretomes)	Comprises putative secretory proteins in 158 fungal/oomycete genomes (208,883 proteins) identified using a three-layer hierarchical identification rule based on nine prediction programs	http://fsd.snu.ac.kr/
		Choi et al., 2010
FSRD (Fungal stress response database)	Incorporates 1985 fungal stress response proteins with verified physiological function(s) and their orthologs identified and annotated in 28 species including human and plant pathogens, as well as important industrial fungi	http://internal.med.unideb.hu/fsrd
		Karányi et al., 2013
PHI-base (Pathogen–host interaction database)	Provides catalogs of experimentally verified pathogenicity, virulence, and effector genes from bacterial, fungal, and oomycete pathogens, which infect human, animal, plant, insect, fish, and fungal hosts	http://www.phi-base.org/
		Winnenburg et al., 2008
FunSecKB (The Fungal Secretome KnowledgeBase)	Provides secretory proteins identified from all available fungal protein data in the NCBI RefSeq database. The secreted proteins were identified using several computational tools	Lum and Min, 2011
		http://proteomics.ysu.edu/secretomes/fungi.php
FunSecKB2 (Fungal protein subcellular location knowledgebase)	Provides an improved and updated version of the fungal secretome and subcellular proteome, i.e., protein subcellular location, knowledgebase	Meinken et al., 2014
		http://proteomics.ysu.edu
Secretool	Secretool comprises a group of web tools that enable secretome predictions	Cortázar et al., 2014
		http://genomics.cicbiogune.es/secretool


Another useful database for CSEPs is FunSecKB, which hosts fungal secretomes identified using six different prediction tools (Lum and Min, 2011). The improved version of FunSecKB comprises about two million proteins covering over 200 fungal species (Meinken et al., 2014). This massive data has enabled to answer several questions regarding the frequency and distribution of secretory proteins in fungi. For instance, Meinken et al. (2014) have observed that fungi with a biphasic lifestyle, such as the hemibiotroph *M. grisea*, have a larger proportion of secreted proteins compared to strict biotrophs or facultative parasites. In general, the size of the secretome is highly correlated with the total size of the proteome.

The accuracy of computation prediction always depends upon functionally validated data used for the training of prediction tools. The mere use of a larger number of tools is not sufficient to achieve higher sensitivity and accuracy. In this context, manual curation and the continuous use of the growing number of experimentally validated protein database should lead to more accurate predictions. In an effort to develop a library of fungal stress response database (FSRD), about 2000 publications, sorted systematically from the PubMed entries, have been used to obtain and define over 2000 stress-related proteins in fungi (Karányi et al., 2013). For the FSRD, care has been taken to avoid including proteins labeled as putative (identified based strictly with computational tools) and to include only genuine proteins characterized experimentally. In spite of this screening procedure, a homology-based search led to the identification of over 29,000 orthologs in 28 fungal/oomycete species (Karányi et al., 2013). Similarly, *in silico* identification of small secretory proteins with several tools, followed by manual curation and homology-based search has identified 1184 and 1066 CSEPs respectively in *Melampsora larici-populina* and *Puccinia graminis* (Duplessis et al., 2011). Considering that, in well-studied fungi such as *Ustilago maydis*, functional studies through gene knockout have identified less than 100 CSEPs (Kämper et al., 2006), it appears that the strategy of identification of homologs using manually verified list of CSEPs, where over 1000 CSEPs per species are predicted, greatly overestimates the number of *bona fide* CSEPs. Therefore, to avoid the identification of false positives, more computational filters should be applied. In this context, a pathogen–host interaction database (PHI-base) has been developed based on functionally characterized proteins involved in disease and initiation of host responses (Winnenburg et al., 2008). The PHI-base initially comprised 405 experimentally verified proteins related to pathogenicity, virulence, and effectors belonging to 54 fungal and oomycete pathogens (Winnenburg et al., 2008). The current version of PHI-base (v 3.6) now comprises about 3000 genes from 4000 interactions, and 160 species including 103 plant pathogens, along with information extracted from 1243 high quality publications (Urban et al., 2014). Such manual curation process and use of experimental studies should be considered along with computational tools to improve the prediction of functional effector proteins.

## Genome-Wide Identification of Candidate Secretory Proteins (CSEPS)

Recent advances in computational tools have made it easier to perform genome-wide identification of CSEPs. However, this approach can often be overlooked considering that several databases hosting predicted secretomes in 100s of fungal and oomycete species are now easily accessible. An obvious drawback to relying on this information is that most of the databases only offer a listing of the secreted proteins with no further characterization of their function or possible role as CSEPs (**Table 4**). Moreover, genome-wide studies provide a better understanding of the distribution and organization of CSEPs within a given species. The characterization of CSEPs in *U. maydis* represents a very good example of the importance of genome-wide analysis. Following whole genome sequencing of *U. maydis*, 426 secretory proteins were identified, 70% of which were annotated with unknown function (based on homology search; Kämper et al., 2006). Of particular importance, most of the *U. maydis* secreted proteins were found to be present in clusters with 3–26 genes per cluster. Knockout of specific genes or clusters allowed a precise identification of about 50 secreted proteins that were involved in pathogenesis (Kämper et al., 2006). In a comparative analysis with other pathogenic Ustilaginales and *Pseudozyma flocculosa*, a non-pathogenic Ustilaginale with biocontrol properties, whole-genome-sequencing revealed a higher conservation of virulent secreted proteins in the three pathogens and a near complete loss in *P. flocculosa* (Lefebvre et al., 2013). In depth analysis of *P. flocculosa* genome revealed that predicted secreted proteins were nearly the same in both *P. flocculosa* and *U. maydis* genome and that the total number of clusters and gene organization of secreted proteins were also quite similar. This approach was thus extremely useful in not only corroborating the secreted proteins involved in virulence in *U. maydis* but also in identifying potential factors involved in the biocontrol properties of *P. flocculosa*. For instance, the presence of two NPP1-containing proteins in the secretome of *P. flocculosa*, absent in all pathogenic Ustilaginales, offers good targets to understand its elusive mode of action. Other striking features, such as introns per gene, have been observed to vary considerably between the two groups (Lefebvre et al., 2013). The role of intron frequency in the structural and functional attributes of genomes has already been suggested in several fungal and plant genomes (Torriani et al., 2011; Deshmukh et al., 2015). Similarly, in addition to the presence of effectors, many other genomic features like GC content, codon bias, gene gain-loss, and in-depth analysis of gene families can be addressed with genome-wide analyses.

## Overview of Candidate Secretory Effector Proteins in Biotrophs and Hemibiotrophs

The biotrophic fungus *U. maydis* is arguably one of the best model pathogens for the study of host–pathogen interactions and molecular mechanisms involved in pathogenesis (Kämper et al., 2006). Its well-annotated genome, and advanced tools for transformation and genome manipulation make it suitable for functional characterization of putative effectors (Kämper et al., 2006; Schuster et al., 2015). In fact, the effector Pep1 is one of the best studied virulence-related proteins for its role in the *U. maydis*-maize interaction. Pep1 inhibits plant peroxidases and suppresses the primary immune response by preventing the oxidative burst. The initial colonization of biotrophs requires a suppression of the immune response in order to interface with its host and acquire nutrients. It has been observed, with confocal microscopy, global expression profiling and metabolic profiling, that *U. maydis* will initially up-regulate defense-response related genes, but, after penetration, will down-regulate the early response genes and also induce genes associated with suppression of cell death (Doehlemann et al., 2008). In mutant *U. maydis* strains with *pep1* gene deletion, no down-regulation of the early response genes was observed (Doehlemann et al., 2009). *U. maydis* was also found to induce genes involved in the synthesis of jasmonic acid but to repress salicylic acid synthesis, a typical response generally observed with biotrophs. Such response was not observed in *U. maydis* Pep1 deletion strain (Doehlemann et al., 2009). Recently, Hemetsberger et al. (2015) identified Pep1 orthologs in genomes of related smut species and performed functional characterization of orthologs by heterologous expression in *U. hordei* and *Hordeum vulgare*. Heterologous expression of Pep1 in *U. hordei* conferred a higher virulence to the mutant strain compared to the wild type. Conversely, heterologous expression of Pep1 in *H. vulgare* was found to increase its susceptibility against the powdery mildew fungus *Blumeria graminis* f. sp. *hordei*, a completely different pathosystem than the maize-*U. maydis*. This suggests the functional conservation of the Pep1 effector across and against different monocots. The high level of sequence conservation suggests the pivotal role of Pep1-like effectors in the pathogenicity of biotrophic fungi. The functional redundancy of Pep1-like effectors has also been observed in pathogens of diverse hosts, both monocots and dicots (Hemetsberger et al., 2015).

Because of their combined biotrophic and necrotrophic lifestyles, hemibiotrophs also produce effectors to suppress early defense responses and maintain their host alive by preventing cell death. At later stages of infection, hemibiotrophs are reported to produce necrotrophic effectors that kill the host. For instance, *P. infestans* secretes AVR3a from its haustoria during the early biotrophic infection stages that suppress cell-death (Whisson et al., 2007). Later in the necrotrophic stages, AVR3a is found to be down-regulated, while INF1 and Nep1-like effectors are secreted, which helps the pathogen to switch from a biotrophic to a necrotrophic stage (Kanneganti et al., 2006).

## Effectors in Bacteria, Nematodes, and Insects

Compared to fungi and oomycetes, bacteria have received considerably more attention with respect to understanding the role of effectors in pathogenicity. Progress has been achieved mostly with the characterization of effectors in gram-negative bacteria that deliver effectors into the host cell by type III (T3SS) or type IV secretion systems (Angot et al., 2007). The whole genome sequencing of 1000s of bacterial isolates and identification of effectors have been used to develop effective computational tools for their prediction (**Table 5**). As a matter of fact, the tools for bacterial effector identification seem more accurate compared to those for fungal effectors. Recently, Teper et al. (2015) used a machine learning algorithm based on 79 features differentiating effector proteins from non-effector proteins to identify novel effectors. The features used for the development of the machine learning approach include several characteristics such as genomic proximity to other effectors, GC content, differential conservation among phytopathogens that do or do not encode a T3S system, amino acid composition at the N-terminus and in the entire protein, T3S-dependent regulation, homology to known T3S effectors of animal- and plant–pathogenic bacteria and similarity to host proteins. After validation of candidate effectors identified in the first round of machine learning, new information is incorporated for the second round of analysis (Teper et al., 2015). Such self-evolving computational approach would also be helpful to identify CSEPs in fungal genomes leading to the identification of more realistic and manageable numbers.

**Table 5:** Features of databases and tools available for the effectors, secreted proteins, and virulence factors identified in bacterial genomes.

Database (specificity)	Important features	Reference/website
AtlasT4SS [Database for type IV secretion system (T4SS) related proteins]	Describes a large number of proteins related to the type IV secretion system reported in Gram-negative, Gram-positive bacteria, and Archaea	Souza et al., 2012
		http://www.t4ss.lncc.br/
DBSecSys (Database of *Burkholderia mallei* Secretion Systems)	Provides a database for computationally predicted bacterial secretion system proteins and their host factors	Memi et al., 2014
LAB-Secretome [Database for the extracellular and surface-associated proteins of Lactic Acid Bacteria (LAB)]	Provides a database comparison of the secretomes from 26 sequenced LAB genomes	Zhou et al., 2010
		www.cmbi.ru.nl/lab_secretome/
Mycosec (A database for signal peptide bearing genes of *mycobacterium)*	Provides computationally predicted signal peptides in the 21 different strains of *Mycobacterium* genome	Sen, 2011
		www.bicnbu.in/mycosec/
SecReT4	Provides a web-based bacterial type IV secretion system resource. T4SSs are versatile assemblages that promote genetic exchange and/or effector translocation with consequent impacts on pathogenesis and genome plasticity	Bi et al., 2012
		http://db-mml.sjtu.edu.cn/SecReT4/
BEAN (Bacterial effector analyzer)	Provides an integrated web resource to predict, analyze, and store type III secreted effectors (T3SEs)	Dong et al., 2013
		http://protein.cau.edu.cn:8080/bean/.
BPBAac (Bacterial type III secreted protein identifier)	Provides a tool that was developed and trained using Support Vector Machine (SVM) based on the Aac feature extracted using a Bi-profile Bayes model. BPBAac classifies T3S and non-T3S proteins very effectively	Wang et al., 2011
		http://biocomputer.bio.cuhk.edu.hk/softwares/BPBAac.
SIEVE Server (Web tool for prediction of type III secreted effectors)	Provides computational prediction of type III and IV secreted effectors in gram-negative bacteria	Samudrala et al., 2009
		http://www.sysbep.org/sieve/
T3SEdb [Database for effectors of Type III secretion system (T3SS)]	Provides keyword as well as sequence searches. More than 171 clusters of T3SEs have been detected based on sequence identity comparisons	Tay et al., 2010
		http://effectors.bic.nus.edu.sg/T3SEdb/index.php
T3SS effector prediction tool	Presents a signal prediction method together with comprehensive survey of potential type III secretion system effectors extracted from 918 published bacterial genomes	Lower and Schneider, 2009
		http://www.modlab.org/


Plant pathogenic nematodes are mostly obligate parasites and depend on living host cells for nutrition. The plant response to nematode presence is genetically similar to the one observed with fungal and bacterial pathogens. Gene for gene evolution is well-documented in the case of nematode resistance and several *Avr* genes and corresponding *R* genes are known (Woo et al., 2014; Kadam et al., 2015; Vuong et al., 2015). Nematodes release degrading enzymes and peptides that mimic plant hormones into the apoplast to make feeding sites by modifying the host cells. The nematode proteins are secreted from specific glands and those are key for the pathogenesis process, in a manner very similar to that observed with the bacterial and fungal effector systems (Mitchum et al., 2013). As a matter of fact, nematode effectors may have evolved after horizontal transfer from bacteria and fungi (Haegeman et al., 2011). Presently very little is known about the specific characteristics of nematode effectors, and as a result, reliable computational tools are more limited for CSEP prediction.

Plant–insect interactions are also being investigated in view of the current understanding of effectors in bacterial and fungal organisms (Stuart, 2015). There are several *Avr* and *R* genes known to dictate plant–insect interactions, and most of these fit well in the gene for gene concept. This suggests the likelihood of molecular mechanisms similar to those found in fungal/bacterial effectors. As with nematodes, horizontal gene transfer from bacteria and fungi has been observed in insects, thereby suggesting a similar process of effector acquisition (Husnik et al., 2013). Plants recognize insects by herbivore-associated molecular patterns (HAMP), similar to PAMPs, which induce an immune response. Insect elicitors are secreted through the saliva at the host–insect interface and induce JA, ethylene and SA biosynthesis, as well as the reactive oxygen burst (Wu and Baldwin, 2010). Such insect recognition and plant response has been observed in *Arabidopsis* in response to proteins present in the green-peach aphid saliva (De Vos and Jander, 2009).

## Concluding Remarks

The rapidly increasing availability of fungal genomes and functionally validated effectors has provided opportunities to improve CSEP identification in many fungal pathogens. In turn, this has led to the development of a large number of computational tools and pipelines to study CSEPs. Given that each tool or pipeline has its own advantages and limitations, the analytical path proposed in this review (**Figure 2**) offers a good balance between computational prediction and effector functionality.

Our review also highlights the need to increase the prediction efficiency of functional secreted proteins by continuously fine-tuning tools with every newly characterized effector. In this context, approaches based on machine learning that can integrate all the information generated through phenotypic and genomic data in a very systematic manner will be helpful in improving identification of effectors. In addition, considering that effectors evolve rapidly through gene-for-gene interactions, comparative genome sequencing data analysis can provide useful insights with respect to CSEP identification, origin, functionality, and important structural features. For instance, secondary and tertiary structure information, gene expression data, and information about gene and genomic organization are likely to increase the accuracy with which effectors are identified in fungi and other organisms. Most of the available pipelines and automated servers do not currently integrate such data. Combining available pipelines with the ever increasing structural, genomic and transcriptomic data will lead to a better prioritization strategy where the most promising effectors can be rapidly targeted for future analyses aimed at a better understanding of pathogenesis processes in plant–pathogen interactions.

## Author Contributions

HS, RD, RB compiled the data, draw the conclusions and wrote the Manuscript. RB designed and supervised the research.

## Conflict of Interest Statement

The authors declare that the research was conducted in the absence of any commercial or financial relationships that could be construed as a potential conflict of interest.

## Supplementary Material

**FIGURE S1 | Flowchart of analytical tools implemented in the Secretool (http://genomics.cicbiogune.es/SECRETOOL/Secretool.php) that was used for the identification of candidate secretory proteins in different plant fungi/oomycetes in Table 2**.
