# Supplementary material for: Computational Prediction of Effector Proteins in Fungi: Opportunities and Challenges
Source: Front Plant Sci. 2016 Feb 12;7:126. doi: 10.3389/fpls.2016.00126 (PMC4751359; doi:10.3389/fpls.2016.00126)
Supplement: Supplementary file 2 [file Table_1.DOCX]

**Table S1.** Databases used to retrieve and analyze protein sequences of fungi and oomycete species in Table 3.

| Sr. No | **Species^†^** | **Total Proteins** | **Database** |
| --- | --- | --- | --- |
| 1 | *Leptosphaeria maculans* | *12469* | *http://genome.jgi.doe.gov/Lepmu1/Lepmu1.home.html* |
| 2 | *Magnaporthe oryzae* | *12755* | *http://fungi.ensembl.org/Magnaporthe_oryzae/Info/Index* |
| 3 | *Ustilago maydis* | *6522* | *http://fungi.ensembl.org/Ustilago_maydis/Info/Index* |
| 4 | *Puccinia graminis* f. sp. *tritici* | *15979* | *http://fungi.ensembl.org/Puccinia_graminisug99/Info/Index* |
| 5 | *Cladosporium fulvum* | *14127* | *http://genome.jgi.doe.gov/Clafu1/Clafu1.home.html* |
| 6 | *Fusarium oxysporum* | *17696* | *http://fungi.ensembl.org/Fusarium_oxysporum/Info/Index* |
| 7 | *Mycosphaerella graminicola* | *10933* | *http://genome.jgi.doe.gov/Mycgr3/Mycgr3.home.html* |
| 8 | *Colletotrichum graminicola* | *12020* | *http://fungi.ensembl.org/Colletotrichum_graminicola/Info/Index* |
| 9 | *Blumeria graminis* | *6470* | *http://fungi.ensembl.org/Blumeria_graminis/Info/Index* |
| 10 | *Alternaria brassicicola* | *10688* | *http://genomeportal.jgi.doe.gov/Altbr1/Altbr1.home.html* |
| 11 | *Pyrenophora tritici-repentis* | *12169* | *http://fungi.ensembl.org/Pyrenophora_triticirepentis/Info/Index* |
| 12 | *Phytophthora_infestans* (Oomycete) | *18140* | *http://protists.ensembl.org/Phytophthora_infestans/Info/Index* |

**Table S2.** Effect of E-value cut-off on the number of different motifs identified in classically secreted proteins of *Magnaporthe* *oryzae* and *Phytopthora* *infestans*

| **E-value** | **RXLR** | **DEER** | **RXLX[EDQ]** | **RXLX** | **[KRHQSA][DENQ]EL** | **Y/W/XC** | **RSIVEQD** |
| --- | --- | --- | --- | --- | --- | --- | --- |
| *Magnaporthe oryzae* | | | | | | | |
| 1.00E-003 | 184 | 80 | 143 | 159 | 119 | 188 | 127 |
| 1.00E-004 | 43 | 2 | 22 | 19 | 16 | 43 | 27 |
| 1.00E-005 | 3 | 0 | 1 | 5 | 0 | 7 | 3 |
| *Phytophthora* *infestans* | | | | | | | |
| 1.00E-003 | 237 | 117 | 133 | 169 | 86 | 152 | 106 |
| 1.00E-004 | 74 | 22 | 16 | 15 | 10 | 16 | 17 |
| 1.00E-005 | 0 | 0 | 2 | 2 | 0 | 4 | 0 |
